# A novel method for examining autistic children’s comprehension of individual words produced within delayed echolalia: a proof-of-concept pilot study

**DOI:** 10.3389/fpsyg.2025.1681076

**Published:** 2025-11-04

**Authors:** Janine Mathée-Scott, Grace Corrigan, Emily Lorang, Zachary Hesse, Jennifer Johnson, Courtney E. Venker

**Affiliations:** ^1^Department of Speech Pathology and Audiology, Marquette University, Milwaukee, WI, United States; ^2^Department of Communicative Sciences and Disorders, Michigan State University, East Lansing, MI, United States

**Keywords:** autism, echolalia, language processing, receptive language, language development, language sample analysis

## Abstract

Delayed echolalia, or the repetition of previously heard speech, is often observed in the expressive language of autistic children. Relatively little is known about how the production of delayed echolalia fits within the overall picture of autistic children’s language ability, including receptive language. To date, no empirical studies have tested autistic children’s comprehension of individual words within their delayed echoes. The present study aimed to establish proof-of-concept for a novel method of examining children’s comprehension of individual words that they produce in their own delayed echoes. Using natural language sampling combined with parent report, we identified instances of delayed echolalia in two young autistic children. We then employed eyegaze methods (i.e., Looking-While-Listening) to test children’s comprehension of individual target words derived from their delayed echoes. Preliminary results revealed that two autistic participants demonstrated comprehension of individual words that they produced in delayed echoes in two different carrier phrases and as single words (*p*’s < 0.001). These findings suggest that it is feasible to employ eyegaze methods to test autistic children’s comprehension of the individual words within their own delayed echoes.

## 1 Introduction

Echolalia refers to the repetition of previously heard speech (Cohn et al., 2022; Luyster et al., 2022). This repetition can be either immediate or delayed. While echolalia is a common feature of autistic language, it is not unique to autism, as it has also been observed in neurotypical language development (Tager-Flusberg and Calkins, 1990), as well as other populations, including adults with aphasia (Schuler, 1979). There are varying definitions of echolalia in the literature (Blackburn et al., 2023; Cohn et al., 2022; Luyster et al., 2022; Stiegler, 2015). Prevalence estimates of echolalia in autism vary widely, with a recent systematic review by Sutherland et al. (2024) finding a range of estimates from 25% to 91% in studies of echolalia in autistic children. Views about how echolalia fits within the overall language profiles of autistic children have evolved over the past several decades. While some scholars view echolalia simply as a repetitive behavior or “vocal stereotypy” (Ahearn et al., 2007; Shawler et al., 2020), others view it as a meaningful form of expressive communication (Prizant and Rydell, 1984; Sterponi and Shankey, 2014). Recently, Dinello and Gladfelter (2025) conducted a scoping review of interventions related to echolalia, finding that historically, interventions overwhelmingly aimed to reduce autistic children’s use of echolalia. However, recent findings suggest that it is both communicatively valuable and has been identified as an aspect of autistic identity and communicative agency (Cohn et al., 2022, 2024; Donaldson et al., 2023). Thus, the field’s understanding of the role of echolalia as a part of some autistic individuals’ expressive language has evolved significantly over the past several decades.

Echolalia is not entirely unique to autism; it appears in typical language development, though tends to decrease as children progress in language development (Fay, 1967; Howlin, 1982). However, descriptions of echolalia can be traced back to the earliest descriptions of autism itself (Kanner, 1943). While previous descriptions and intervention recommendations primarily pathologized the use of echolalia in autistic individuals (Dinello and Gladfelter, 2025), more recently, echolalia has become increasingly celebrated as a positive part of autistic identity and experience (Cohn et al., 2024). This shift has been supported by work that has elucidated the pragmatic functions of echolalia (Prizant and Rydell, 1984; Sterponi and Shankey, 2014), as well as by the broader neurodiversity movement, which aims to improve societal views of autistic traits, including echolalia (Bottema-Beutel et al., 2023; Gaddy and Crow, 2023). Researchers have recently endeavored to create methods for characterizing unconventional forms of language in autistic children, including echolalia (Gladfelter and VanZuiden, 2020; Luyster et al., 2022; Maes et al., 2024), although there remains little consensus as to the appropriate approach to doing so, and as to the interpretation of how this language fits within the broader expressive language profiles of autistic children.

To date, there has been a paucity of research on the receptive language component of echolalia (but see Prizant and Rydell, 1984). That is, how much of the language that children repeat in delayed echolalia do they understand? This question carries both theoretical and clinical significance. In recent years, a clinical approach, known as the Natural Language Acquisition (NLA) protocol, has gained wide clinical popularity and is based on the hypothesis that autistic children who produce delayed echolalia process language in a “gestalt” manner (Blanc et al., 2023). This hypothesis, termed “Gestalt Language Processing,” posits that children who repeat chunks of language do not initially process the individual words within those chunks, but rather process these chunks as “unanalyzed wholes” (Blanc et al., 2023; Haydock et al., 2024). To date, this assumption has not been empirically tested (Bryant et al., 2024; Hutchins et al., 2024; Venker and Lorang, 2025). Despite a lack of empirical evidence, a set of intervention recommendations regarding the language input adults provide has been made regarding the language input adults provide, based on the assumption that children may not understand the individual words produced within their echoes, at least at certain proposed stages of development (Blanc et al., 2023).

In light of these evolving and varied views of echolalia, there is a critical need for increased empirical and clinical understanding of how delayed echolalia fits into the overall picture of autistic children’s language. In particular, relatively little is known about how echolalia should be considered with respect to receptive language. To this end, it would be clinically and theoretically important to know whether autistic children comprehend the individual words within the delayed echoes they produce. Prior research has validated the use of eyegaze methods for assessing children’s language processing and comprehension, including for children on the autism spectrum (Arunachalam et al., 2024; Haebig et al., 2015; Pomper et al., 2021; Venker et al., 2013; Venker and Kover, 2015). One of these studies (Arunachalam et al., 2024) validated an approach in which this eyegaze task was personalized to children’s own interests, paving the way for future work to personalize language processing tasks to each child. Additionally, some research groups have described methods for identifying echolalia in children, including identifying instances of echolalia in language samples (Gladfelter and VanZuiden, 2020; Luyster et al., 2022; Maes et al., 2024).

The aim of the present study is to establish proof-of-concept for employing established eyegaze language processing methods (e.g., the Looking-While-Listening (LWL) protocol; Fernald et al., 2008) for testing children’s comprehension of the individual words they produce in delayed echoes. This novel method combines aspects of the methods described above to examine comprehension of words within children’s own delayed echoes. We tested this novel method by personalizing eyegaze tasks to two autistic children, including individual words from each child’s own delayed echoes and probing their comprehension of those words. We hypothesized that children would demonstrate comprehension of individual words that they produced in their own delayed echoes.

## 2 Materials and equipment

### 2.1 Language sampling

Minimal equipment is required to collect caregiver-child language samples from which to identify instances of delayed echolalia. Caregivers and children typically play with a set of toys (e.g., farm set, car, and truck toys) in a laboratory setting, or with their own toys if administered in-home (Barokova and Tager-Flusberg, 2020; Plate, 2025). A video camera and audio recorder is required for in-lab administration, or a high-quality web-camera along with an audio recorder for virtual (i.e., in-home) administration.

### 2.2 Eyegaze experiment

The physical equipment required to administer LWL tasks includes a television, video camera, experimental computer, chair, and a sound-attenuated experimental space. Some research groups have used automatic eyetracking for this type of experiment, which would require an automatic eyetracker, such as the Tobii X2-60. We elected to hand-code the data for the present study, given that prior work has demonstrated better data retention using hand-coding (Venker et al., 2019a,b). Hand-coding of eyegaze data requires a computer and the software program peyeCoder, which is open access (Olson et al., 2020). Experimental administration also requires a software program, such as E-Prime (Psychology Software Tools, Pittsburgh, PA, USA). Stimulus preparation requires Photoshop software (Adobe Inc, 2019) for visual stimuli, and a microphone and Praat software (Boersma, 2001) for recording and editing auditory stimuli.

## 3 Method

### 3.1 Procedure

#### 3.1.1 Standardized assessments

The Autism Diagnostic Observation Schedule, Second Edition (ADOS-2; Lord et al., 2012) was administered by trained laboratory staff to confirm autism diagnosis; scoring was conducted by a research-reliable examiner. Children also participated in the Preschool Language Scales, Fifth Edition (PLS-5) (Zimmerman et al., 2011) and Mullen Scales of Early Learning (Mullen, 1995) to evaluate broader cognitive, developmental and language abilities.

#### 3.1.2 Identifying delayed echolalia

We developed a method for identifying instances of each child’s productions of delayed echolalia using a three-tiered approach: language sampling, consensus coding, and collaboration with parents. Our goal was not to comprehensively identify every instance of delayed echolalia, but rather to identify a subset of utterances which were highly likely to be delayed echoes, so that the individual words within those echoes could be tested for comprehension. Participants and their caregivers engaged in two naturalistic, 10-min play-based language samples in the lab. They were given a set of toys (e.g., farm set, car toys) and instructed to play as they would at home. Both video- and audio-recordings were obtained. In addition to these language samples, we examined audio- and video-recordings from the ADOS-2 for instances of potential delayed echolalia.

Next, we undertook a two-step process for identifying delayed echoes within these samples. First, two research assistants were trained to identify delayed echolalia by reading research articles (Gladfelter and VanZuiden, 2020; Luyster et al., 2022) and discussing video examples. These coders independently reviewed the videos and used intonation and linguistic context (Gladfelter and VanZuiden, 2020; Luyster et al., 2022) to mark utterances as “possible delayed echolalia.” Similar to Gladfelter and VanZuiden’s (2020) approach, prosody was used as one factor for coders to consider when identifying an utterance as echolalic. For example, when the utterance followed a “consistent intonation pattern,” likely resulting from the child imitating the intonation of the original source of the speech (e.g., television), or when the intonational pattern was exaggerated (as outlined in the ADOS-2; Lord et al., 2012). The two coders then compared codes. Only those utterances that both coders had identified as “possible delayed echolalia” were included in the second step. The second step was to confirm that the utterance was a repetition of previously heard speech, via parent report. Parents completed a checklist with laboratory staff over the phone. Parents were asked if they had heard their child repeat each phrase and with what frequency (i.e., Never, Monthly, Weekly, and Daily). Parents were asked to name the source of the phrase if they knew it (e.g., *Daniel Tiger*, *Wheel of Fortune*). Any phrases that the parent did not endorse having heard the child repeat before (i.e., “Never”) were excluded from the final task. Parents were also given the opportunity to add phrases that were commonly observed at home but did not appear in the language samples. Only the subset of utterances that were confirmed by parent report were included as possible targets.

#### 3.1.3 Personalized eyegaze task

Once the delayed echoes had been identified, we examined each child’s list of delayed echoes for potential target words. Possible target words could be nouns or verbs but needed to be imageable for inclusion in the LWL task (see Table 1 for utterances and associated targets). Once targets were identified, we created visual and auditory stimuli. When possible, we used parent input about the source of each phrase to guide the selection of visual stimuli (e.g., in the case of specific characters from television shows such as *Daniel Tiger*). We used Google Image searches and Adobe Photoshop (Adobe Inc, 2019) to create visual stimuli, in which the target object appeared on a solid grey background square. We recorded auditory stimuli using Praat software (Boersma, 2001). For each target word, we recorded three sound tokens. First, in “Find the…” phrases (e.g., “Find the froggy.”), next in “Look at the…” phrases, (e.g., “Look at the froggy.”), and finally as single words, along with an auditory primer (e.g., “Ooh! Froggy,” “Look! Froggy.”). Targets were placed into yoked pairs, such that each target was always presented with the same distractor item, as is standard for LWL tasks (Fernald et al., 2008). The task was structured into three trial types, which were presented sequentially. First, all targets were presented in “Find the…” phrases; next all targets were presented in “Look at the…” phrases; and finally, all targets were presented in single words. Target location was pseudo-randomized, ensuring that for each trial type, target location occurred an equal number of times on the Right and the Left side of the screen, and target location varied throughout the task. We programmed the experiment using E-Prime software (Psychology Software Tools, Pittsburgh, PA, USA). See Tables 2, 3 for each participant’s full task structure.

**Table 1:** Delayed echoes and associated target words.

Participant 1 echoes	Participant 1 target words	Source (if provided)
“Want shapes”	Shapes	Unspecified
“Jump like a froggy”	Jump (V)	Ms. Rachel video
“Jump like a froggy”	Froggy	Ms. Rachel video
“Down the slide”	Slide	Therapy
“I want swings”	Swing	Therapy
“Keepy Uppy”	Keepy Uppy	Bluey (television show)
“Miska Mooska Mickey Mouse, say it with me!”	Mickey Mouse	Mickey Mouse Clubhouse (television show)
“This is a dog”	Dog	Ms. Rachel video
“Peel banana”	Peel (V)	Song
“Peel banana”	Banana	Song
“Want letters”	Letters	Elmo toy
“Want oranges”	Orange	Unspecified

Participant 2 echoes	Participant 2 target words	Source (if provided)
“It lands on the Jackpot!”	Jackpot	Wheel of Fortune (television show)
“Plinko!”	Plinko	The Price is Right (television show)
“When I was a little boy I went to school.”	Boy	Dad
“When I was a little boy I went to school.”	School	Dad
“Mom and Dad will pick you up at the end of the day.”	Mom	Mom/Dad
“Mom and Dad will pick you up at the end of the day.”	Dad	Mom/Dad
“It’s Daniel Tiger’s neighborhood”	Daniel Tiger	Daniel Tiger’s Neighborhood (television show)
“It’s Daniel Tiger’s neighborhood”	Neighborhood	Daniel Tiger’s Neighborhood (television show)
“It’s a beautiful day in the neighborhood”		
“And now, here is the host of Jeopardy, Ken Jennings!”	Ken Jennings	Jeopardy (television show)
“From the Alex Trebek stage at Sony Pictures studios, this is Jeopardy!”	Alex Trebek	Jeopardy (television show)
“From the Alex Trebek stage at Sony Pictures studios, this is Jeopardy!”	Jeopardy	Jeopardy (television show)
“From Sony Pictures studios, it’s America’s game! Wheel of Fortune!”	Wheel of Fortune	Wheel of Fortune (television show)

(V) indicates the target word is a verb. Parents were asked to provide the source of the original utterance (e.g., specific television show) if it was known, parents’ answers are reported.

**Table 2:** Participant 1 task structure.

Trial number	Trial type	Left image	Right image	Auditory stimuli
1	Carrier 1	**Jump**	Peel	“Find jump”
2	Carrier 1	Froggy	**Dog**	“Find the dog”
3	Carrier 1	Keepy Uppy	**Mickey Mouse**	“Find Mickey Mouse”
4	Carrier 1	**Shapes**	Letters	“Find the shapes”
5	Carrier 1	Slide	**Swing**	“Find the swing”
6	Carrier 1	Jump	**Peel**	“Find peel”
7	Carrier 1	**Froggy**	Dog	“Find the froggy”
8	Carrier 1	Shapes	**Letters**	“Find the letters”
9	Carrier 1	**Slide**	Swing	“Find the slide”
10	Carrier 1	**Orange**	Banana	“Find the orange”
11	Carrier 1	Mickey Mouse	**Keepy Uppy**	“Find keepy uppy”
12	Carrier 1	**Banana**	Orange	“Find the banana”
13	Carrier 2	Letters	**Shapes**	“Look at the shapes”
14	Carrier 2	**Jump**	Peel	“Look at jump”
15	Carrier 2	**Banana**	Orange	“Look at the banana”
16	Carrier 2	Dog	**Froggy**	“Look at the froggy”
17	Carrier 2	Keepy Uppy	**Mickey Mouse**	“Look at Mickey Mouse”
18	Carrier 2	**Orange**	Banana	“Look at the orange”
19	Carrier 2	Slide	**Swing**	“Look at the swing”
20	Carrier 2	Shapes	**Letters**	“Look at the letters”
21	Carrier 2	**Slide**	Swing	“Look at the slide”
22	Carrier 2	**Dog**	Froggy	“Look at the dog”
23	Carrier 2	**Keepy Uppy**	Mickey Mouse	“Look at keepy uppy”
24	Carrier 2	Jump	**Peel**	“Look at peel”
25	Single word	Mickey Mouse	**Keepy Uppy**	“Look keepy uppy”
26	Single word	**Froggy**	Dog	“Ooh froggy”
27	Single word	**Banana**	Orange	“Ooh banana”
28	Single word	Peel	**Jump**	“Ooh jump”
29	Single word	**Orange**	Banana	“Look orange”
30	Single word	Froggy	**Dog**	“Look dog”
31	Single word	**Mickey Mouse**	Keepy Uppy	“Ooh Mickey Mouse”
32	Single word	Shapes	**Letters**	“Ooh letters”
33	Single word	Jump	**Peel**	“Look peel”
34	Single word	**Swing**	Slide	“Look swing”
35	Single word	**Shapes**	Letters	“Look shapes”
36	Single word	Swing	**Slide**	“Ooh slide”

Target object in **bold**.

**Table 3:** Participant 2 task structure.

Trial number	Trial type	Left image	Right image	Auditory stimuli
1	Carrier 1	Plinko	**Jackpot**	“Find the Jackpot”
2	Carrier 1	**Mom**	Dad	“Find Mom”
3	Carrier 1	**Alex Trebek**	Ken Jennings	“Find Alex Trebek”
4	Carrier 1	Neighborhood	**Boy**	“Find the boy”
5	Carrier 1	**Daniel Tiger**	School	“Find Daniel Tiger”
6	Carrier 1	Jackpot	**Plinko**	“Find Plinko”
7	Carrier 1	**Ken Jennings**	Alex Trebek	“find Ken Jennings”
8	Carrier 1	Mom	**Dad**	“Find Dad”
9	Carrier 1	**School**	Daniel Tiger	“Find the school”
10	Carrier 1	Jeopardy	**Wheel of Fortune**	“Find Wheel of Fortune”
11	Carrier 1	Wheel of Fortune	**Jeopardy**	“Find Jeopardy”
12	Carrier 1	Boy	**Neighborhood**	“Find the neighborhood”
13	Carrier 2	**Jeopardy**	Wheel of Fortune	“Look at Jeopardy”
14	Carrier 2	School	**Daniel Tiger**	“Look at Daniel Tiger”
15	Carrier 2	**Plinko**	Jackpot	“Look at Plinko”
16	Carrier 2	Daniel Tiger	**School**	“Look at the school”
17	Carrier 2	Ken Jennings	**Alex Trebek**	“Look at Alex Trebek”
18	Carrier 2	**Boy**	Neighborhood	“Look at the boy”
19	Carrier 2	**Wheel of Fortune**	Jeopardy	“Look at Wheel of Fortune”
20	Carrier 2	Alex Trebek	**Ken Jennings**	“Look at Ken Jennings”
21	Carrier 2	**Neighborhood**	Boy	“Look at the neighborhood”
22	Carrier 2	Mom	**Dad**	“Look at Dad”
23	Carrier 2	**Jackpot**	Plinko	“Look at the Jackpot”
24	Carrier 2	**Mom**	Dad	“Look at Mom”
25	Single word	Boy	**Neighborhood**	“Look neighborhood”
26	Single word	**Mom**	Dad	“Ooh Mom”
27	Single word	**Alex Trebek**	Ken Jennings	“Ooh Alex Trebek”
28	Single word	Daniel Tiger	**School**	“Ooh school”
29	Single word	**Wheel of Fortune**	Jeopardy	“Look Wheel of Fortune”
30	Single word	School	**Daniel Tiger**	“Look Daniel Tiger”
31	Single word	**Jackpot**	Plinko	“Ooh Jackpot”
32	Single word	Alex Trebek	**Ken Jennings**	“Ooh Ken Jennings”
33	Single word	Neighborhood	**Boy**	“Look boy”
34	Single word	**Jeopardy**	Wheel of Fortune	“Look Jeopardy”
35	Single word	**Plinko**	Jackpot	“Ooh Plinko”
36	Single word	Mom	**Dad**	“Look Dad”

Target object in **bold**.

The experiment was administered using standard LWL procedures, in which the child sits on their caregiver’s lap and views the experiment on a television screen (see Equipment and Materials). The parent wore opaque sunglasses to prevent any unintentional influence on the child’s looking behaviors. Videos of the child’s face during the task were exported and coded offline without sound by trained coders who were unaware of target location. Coders used Peyecoder software (Olson et al., 2020) and standard coding procedures.

### 3.2 Participants

Participants were two autistic children who had community diagnoses of autism confirmed by trained clinical laboratory staff (see “Standardized assessments”); these children were initially recruited as part of a larger study. Participant 1 (Female) was 40 months of age and Participant 2 (Male) was 58 months of age at the time of the present study. Participant 1 had a standard score of 50 on the PLS-5 auditory comprehension subtest and a standard score of 83 on the PLS-5 expressive communication subtest. Participant 2 had a standard score of 79 on the PLS-5 auditory comprehension subtest and a standard score of 76 on the PLS-5 expressive communication subtest. The study protocol was prospectively approved by the Institutional Review Board at Michigan State University.

### 3.3 Data processing and cleaning

Data were exported from Peyecoder for analysis in R Studio (Version 2024.04.2+764). Similar to prior research (Borovsky and Creel, 2014; Pomper et al., 2021; Mathee-Scott et al., 2022; Venker et al., 2020), we excluded trials in which the child was looking at the screen for less than 50% of the total trial. This resulted in 30 useable trials (of 36 possible) for Participant 1 and 35 useable trials (of 36 possible) for participant 2. We conducted post-hoc critical onset time calculations to determine exact target onset for each trial.

## 4 Results

We examined children’s eye movements during a standard analytical window of 300–1,800 ms after target onset, similar to prior work (Curtis et al., 2023; Fernald et al., 2008; Marchman et al., 2016; Ronfard et al., 2022). First, we calculated participants’ average accuracy (i.e., mean proportion of looks to target vs. nontarget during the analytical window) for each trial type. We then conducted one sample *t*-tests against chance level (equal looks to target vs. distractor) to evaluate whether children’s looks to the target object were significantly above chance which would suggest comprehension of the target words.

### 4.1 Participant 1

**Figure 1:**
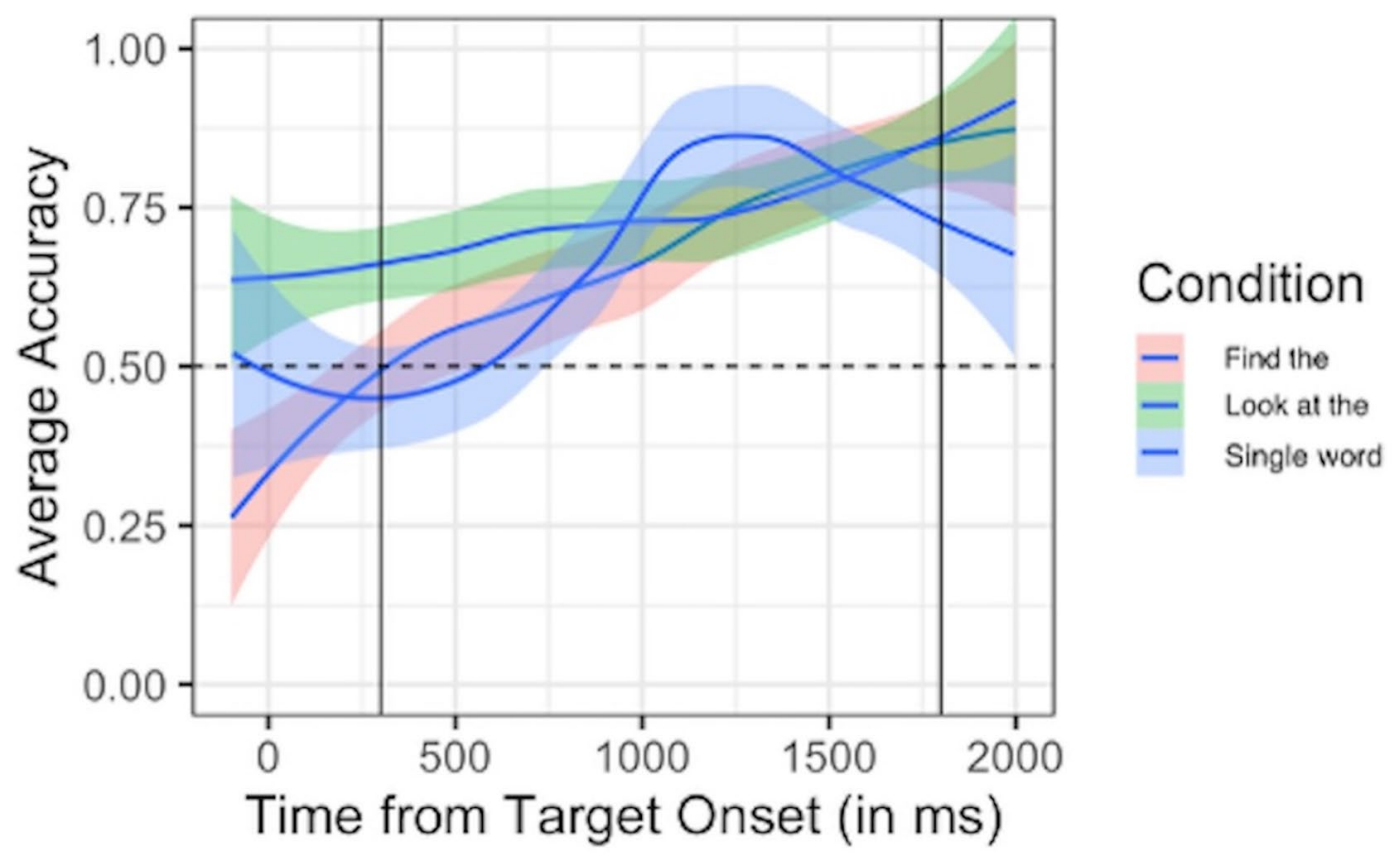
Participant 1 looking behavior. Average accuracy (proportion of looks to target vs. non-target) plotted over time since target onset for each trial type. Carrier 1 (“Find the”) trials are plotted in pink; Carrier 2 (“Look at the”) trials are plotted in green; Single word trials are plotted in blue. Dashed horizontal line indicates chance performance (0.5 proportion of looks to target vs. non-target). Solid vertical lines indicate the analytical window (300–1,800 ms after target onset).

For “Find the…” phrases, Participant 1’s mean accuracy was 69.55%, which was significantly above chance based on *t*-test results (*t* = 8.28, *p* < 0.001). For “Look at the…” phrases, Participant 1 had a mean accuracy of 74.06%, which a *t*-test confirmed was significantly above chance level (*t* = 11.39, *p* < 0.001). For single word trials, Participant 1’s mean accuracy was 68.23%, which a *t*-test confirmed was significantly above chance (*t* = 6.70, *p* < 0.001). Thus, Participant 1 demonstrated comprehension of target words at significantly greater than chance levels in both carrier phrases and single word trials (see Figure 1).

### 4.2 Participant 2

**Figure 2:**
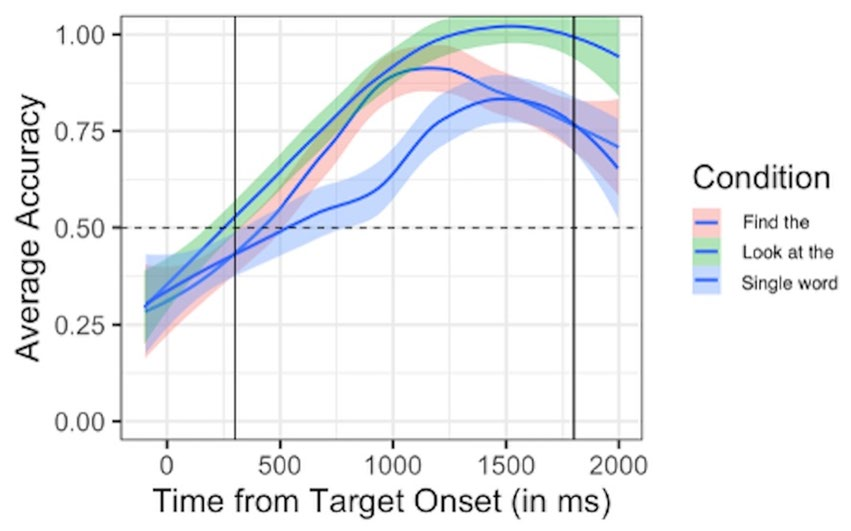
Participant 2 looking behavior. Average accuracy (proportion of looks to target vs. non-target) plotted over time since target onset for each trial type. Carrier 1 (“Find the”) trials are plotted in pink; Carrier 2 (“Look at the”) trials are plotted in green; Single word trials are plotted in blue. Dashed horizontal line indicates chance performance (0.5 proportion of looks to target vs. non-target). Solid vertical lines indicate the analytical window (300–1,800 ms after target onset).

Participant 2’s mean accuracy for “Find the…” phrases was 75.87%. This was significantly greater than chance levels according to *t*-test results (*t =* 12.31, *p* < 0.001). For “Look at the…” phrases, Participant 2 had a mean accuracy of 86.24%, which *t-*tests confirmed was significantly above chance level (*t* = 22.82, *p* < 0.001). Participant 2 had a mean accuracy of 67.26% for single word trials, which was significantly above chance levels (*t* = 7.75, *p* < 0.001). Participant 2 demonstrated comprehension of target words, as evidenced by looks to target significantly above chance levels, in both carrier phrases and as single words (see Figure 2). Thus, there were no clear differences in performance based on whether target words were presented in “Find” phrases, “Look at” phrases, or as single words.

## 5 Discussion

To our knowledge, this is the first evidence that it is feasible to identify instances of delayed echolalia through language sampling and parent report, and to create individualized LWL tasks to include individual, imageable targets from those identified phrases. Additionally, these findings present the first preliminary evidence that autistic children demonstrate comprehension of individual words within their delayed echoes. These findings, if replicated with a larger sample, have important implications for how clinicians and researchers consider delayed echolalia within the overall language profiles of autistic children. Our findings, though preliminary, suggest that autistic children who produce delayed echolalia do process individual words within those phrases as single words, as well as within two different carrier phrases.

These preliminary findings open several exciting opportunities for further scientific inquiry. This work demonstrates the feasibility of creating individualized eyegaze tasks for each participant based on the delayed echolalia they produce. Anecdotally, children appeared to enjoy participating in these tasks, based on smiling, laughing, and in some cases verbalizing (e.g., “I like this show!”). We suspect this is because the tasks were personalized to the children and thus included some of their favorite objects and characters, given that their delayed echoes were in many cases related to their interests (e.g., television shows). Such personalized tasks also may have the potential to increase engagement and motivation, thus providing another methodological advantage. To our knowledge, this is the first study to test children’s comprehension of the words within their own delayed echoes. However, this study builds on prior research that has personalized eyegaze tasks to individual children. Arunachalam et al. (2024) pioneered this approach by testing children’s ability to learn novel words that were and were not related to their focused interests. Similarly, Rothwell et al. (2024) selected targets for a word learning study based on the categories that children were interested in (using parent input) at the group level (e.g., animals). These innovative approaches pushed the field forward, and future research should continue to explore novel methods for examining important research questions using tailored experimental designs.

Future research should continue to refine methods for identifying echolalia, so that it can continue to be studied and better understood. For example, future research should evaluate comprehension of immediate echolalia in addition to delayed echolalia, and for a broader sample of children of varied ages, sociodemographic characteristics, and language and cognitive abilities. Future research might also examine relationships between the linguistic features of children’s delayed echoes and their broader language level. It is notable that our two participants varied in their age and broader language ability, and the complexity of their delayed echoes appeared to mirror this difference as well, with Participant 2 who was older with more advanced overall language abilities producing more complex, longer delayed echoes than our younger Participant 1. Additionally, it may be informative to examine whether there are meaningful differences in children’s comprehension of individual words depending on whether they are presented as single words, in carrier phrases, and which type of carrier phrase. Based on our post-hoc trial-type analyses, for our participants there did not appear to have been a clear effect of trial-type, however future work with more participants might be better positioned to examine this effect.

It is important to note some limitations of the present study. First, we were only able to test children’s comprehension of words within echoes that we could confidently identify using our method combining language sampling, consensus coding, and parent report. This method likely did not comprehensively identify *all* instances of delayed echolalia. Additionally, we were only able to test comprehension of individual words that were imageable, given that the LWL method includes visual representations of target words. Thus, we did not have the ability to test comprehension of every individual word within each child’s delayed echoes. For example, one of our participants used the delayed echo: “I have a surprise for you and you are really gonna like it!” This phrase does not include any words that could be visually represented in a LWL task and thus was not included. As such, we cannot say whether these children would demonstrate comprehension of all of the words represented in their delayed echoes. It is also important to acknowledge that these results are preliminary and cannot yet be generalized to the larger population of autistic children. Rather, these results suggest that this innovative, personalized experimental method is feasible, and future research should continue to test autistic children’s processing of individual words produced in echolalia.

One additional consideration to be acknowledged is that we did not ask parents whether their children’s delayed echoes were verbatim repetitions, or slight variations on the original language. This may be important to consider in future work, given that there are varied definitions of echolalia (Blackburn et al., 2023; Luyster et al., 2022; Stiegler, 2015), with some definitions including occurrences such as self-repetitions and mitigations, or slight variations of the words used from previously heard speech. We used Luyster et al. (2022) “non-generative language” framework to guide our classification utterances as echolalic. In future work, it may be useful to include a portion of the parent consultation which inquires whether the child varies the language within the delayed echo or repeats the phrase exactly as it was previously heard. Investigations of possible comprehension differences between words within these types of utterances may also be informative. There are also limitations to the Looking-While-Listening method that should be acknowledged. First, looking behavior (i.e., children’s looks to the target object) is used as a proxy for comprehension. Second, the method only allows for testing of targets that are imageable. Because of this, we were unable to test comprehension of every word within our participants’ delayed echoes, which would be necessary to get the most complete picture of children’s comprehension of their delayed echoes.

One important feature of the task is that the individual words were tested outside of the delayed echoes in which they were produced. This means that children’s comprehension of the individual words can be separated from any potential associations they have made between the longer phrases and the concepts associated with them. Additionally, it is notable that in two phrases for Participant 1 and in four phrases for Participant 2, there were two target words within the same phrase (e.g., “**jump** like a **froggy**”), which were each tested separately. For Participant 2, there was also a phrase in which the two target words that were derived from the same phrase were yoked together (e.g., “**Mom** and **Dad** will pick you up at the end of the day;” see Tables 2, 3 for task structure). Thus, if the phrase or chunk of language was associated with only one of the images, the child would not have demonstrated accurate looking behavior for both targets. As such, children could not have simply been demonstrating an association between the phonological information from the delayed echo, or “chunk” of language, and the image associated with it, as suggested by the Gestalt Language Processing hypothesis (Blanc et al., 2023; Haydock et al., 2024; Peters, 1983). Rather, we believe our preliminary results serve as evidence for processing of individual words within delayed echolalia, rather than as evidence of a simple association between language chunks and individual concepts or images.

The findings of the present study should also be considered alongside the large body of research on autistic children’s broader language processing. There is a significant body of research demonstrating autistic children’s incremental (i.e., word by word) processing of language more broadly (Bavin et al., 2014, 2016; Prescott et al., 2022; Venker et al., 2019a; Zhou et al., 2019). This research contradicts the claim that autistic children process language in larger “chunks” (i.e., gestalt processing) rather than word-by-word. The preliminary findings of the present study build on this body of research, which is inconsistent with claims that autistic children do not process the individual words within larger chunks of language, and only process these units of language as unanalyzed wholes (Peters, 1983). Though additional work is needed, in view of the present study, we propose that clinicians should take caution before implementing clinical recommendations based on the assumption that autistic children who produce delayed echolalia do not process the individual words within those phrases.

## Data Availability

The raw data supporting the conclusions of this article will be made available by the authors, without undue reservation. Supplementary visual stimuli is not readily available due to copyright restrictions, but can be made available upon reasonable request.
